# ADRB2-Targeting Therapies for Prostate Cancer

**DOI:** 10.3390/cancers11030358

**Published:** 2019-03-13

**Authors:** George Kulik

**Affiliations:** 1 Department of Cancer Biology, Wake Forest University Health Sciences, Medical Center Blvd, Winston-Salem, NC 27157, USA; gkulik@wakehealth.edu or gkulik@alfaisal.edu; 2 Department of Life Sciences, Alfaisal University, Riyadh 11533, Saudi Arabia

**Keywords:** β-2 adrenergic receptor (ADRB2), cAMP-dependent protein kinase (PKA), prostate cancer, propranolol, apoptosis, clinical trial, BCL-2-associated death promoter (BAD), myeloid cell leukemia 1 (MCL-1)

## Abstract

There is accumulating evidence that β-2 adrenergic receptor (ADRB2) signaling contributes to the progression and therapy resistance of prostate cancer, whereas availability of clinically tested β-blocker propranolol makes this pathway especially attractive as potential therapeutic target. Yet even in tumors with active ADRB2 signaling propranolol may be ineffective. Inhibition of apoptosis is one of the major mechanisms by which activation of ADRB2 contributes to prostate cancer pathophysiology. The signaling network that controls apoptosis in prostate tumors is highly redundant, with several signaling pathways targeting a few critical apoptosis regulatory molecules. Therefore, a comprehensive analysis of ADRB2 signaling in the context of other signaling mechanisms is necessary to identify patients who will benefit from propranolol therapy. This review discusses how information on the antiapoptotic mechanisms activated by ADRB2 can guide clinical trials of ADRB2 antagonist propranolol as potential life-extending therapy for prostate cancer. To select patients for clinical trials of propranolol three classes of biomarkers are proposed. First, biomarkers of ADRB2/cAMP-dependent protein kinase (PKA) pathway activation; second, biomarkers that inform about activation of other signaling pathways unrelated to ADRB2; third, apoptosis regulatory molecules controlled by ADRB2 signaling and other survival signaling pathways.

## 1. Introduction

Since androgen ablation was demonstrated as efficient therapy for advanced prostate cancer [1] substantial efforts have been focused on development of drugs that target androgen receptor (AR) signaling. Indeed, androgen ablation by an inhibitor of androgen biosynthesis, abiraterone acetate, and by enzalutamide, an AR antagonist that prevents nuclear translocation and chromatin binding, extend survival of prostate cancer patients. Still, despite improved AR axis targeting, the disease progresses to castration resistant prostate cancer (CRPC), which is refractory to existing therapies [2,3]. Comprehensive genomic profiling of advanced prostate tumors demonstrated substantial genetic inter-tumor and intra-tumor heterogeneity that translates into a wide range of diverse signaling mechanisms that contribute to prostate cancer pathophysiology [4,5]. For example, a constitutive activation of phosphatidyl inositol 3 kinase (PI3K) pathway, mitogen-activated protein kinase kinase/mitogen-activated protein kinase MEK/ERK pathway and Wnt/beta catenin pathway have been reported to sustain survival and proliferation of androgen-independent prostate cancer cells [6]. Therefore, a uniform therapeutic approach focused on androgen signaling has to be supplemented or even replaced by “personalized” approach that targets signaling pathways specific for a given tumor [7,8]. 

Recently signaling via ADRB2/PKA module has been connected with prostate cancer progression and therapy resistance [9]. This minireview discusses how information on the mechanisms by which ADRB2 activation inhibits apoptosis can be used to guide clinical trials of ADRB2 antagonist propranolol as potential life-extending therapy for prostate cancer. 

## 2. ADRB2 Signaling in Prostate Cancer Progression

ADRB2 belong to superfamily A of seven-transmembrane G protein-coupled receptors (GPCRs) activated by epinephrine (Epi) or norepinephrine (NE) [10]. Ligand binding increases intrinsic guanine nucleotide exchange factor (GEF) activity of ADRB2 that converts associated Gα into guanosine triphosphate (GTP)-bond state and triggers dissociation of Gα-GTP and Gβγ subunits of heterotrimeric G proteins [11,12]. Upon dissociation from Gβγ, a Gα_s_-GTP subunit binds to and activates adenylyl cyclase that generates cAMP. There are three main effectors of cAMP: PKA, the guanine-nucleotide-exchange factor EPAC and cyclicnucleotide-gated ion channels [13]; whereas Gβγ subunits bind G-protein coupled receptor kinases (GRK) that phosphorylate the cytoplasmic loop 4 of ADRB2. Phosphorylation by GRK creates binding sites for β-arrestins, scaffold proteins that prevent interaction of ADRB2 with Gα_s_ and down-regulate ADRB2/cAMP/PKA signaling. In addition, β-arrestin-ADRB2 complex serves as scaffold to activate several downstream signaling pathways including MEK/ERK, PI3K, SRC and to drive ADRB2 endocytosis [14]. 

A substantial amount of information about ADRB2 signaling was obtained from experiments with synthetic ligands that can selectively activate or inhibit specific receptor types. For example, isoproterenol selectively activates ADRB2 receptors whereas ICI118,551, atenolol, and propranolol act as inverse agonists by preventing activation of ADRB2/PKA signaling pathway by catecholamines. ICI118,551 selectively inhibits ADRB2, atenolol binds ADRB1 with 15-fold higher affinity comparing to ADRB2, whereas propranolol binds both ADRB1 and ADRB2 with comparable affinity [15,16,17,18].

ADRB2 are expressed in luminal prostate epithelial cells and prostate cancer cells [19,20,21,22]. They can be activated by systemic Epi secreted by adrenal medulla in response to psychoemotional, physical or metabolic stresses and by NE locally secreted by sympathetic nerve terminals that binds ADRB2 with approximately 100-fold lower affinity than Epi. In addition, NE and Epi can be secreted by lymphocytes, macrophages and neutrophils in prostate tumor microenvironment [23,24,25].

In mouse models of prostate cancer activation of ADRB2/PKA pathway by systemic Epi elevated in response to psychoemotional stress or by injections of Epi diminished the efficacy of androgen ablation and cytotoxic therapies, whereas infusion of NE facilitated the development of metastases. Conversely, ADRB2/PKA pathway inhibition with beta blockers (ICI118,551, propranolol) attenuated effects of stress and of injected NE [26,27]. Increased MCL-1 expression and BAD phosphorylation were identified as target molecules responsible for apoptosis inhibition in prostate cancer cells by ADRB2/PKA pathway, whereas the effectors responsible for accelerated migration await further investigation [28,29]. 

MCL-1 and BAD belong to a family of BCL2 proteins that play a central role in apoptosis by regulating mitochondrial outer membrane permeability (MOMP) and the release of apoptosis-inducing proteins (cytochrome c, second mitochondria-derived activator of caspase (SMAC) and apoptosis inducing factor (AIF)) from mitochondria. Based on their role in regulating MOMP and presence of conserved BCL2-homology (BH) domains, BCL2 proteins family is divided into 3 subfamilies: multidomain anti-apoptotic proteins, multidomain pro-apoptotic proteins, and BH3-only pro-apoptotic proteins. Multi-domain pro-apoptotic proteins such as BAK and BAX (with BH1-3 domains) can oligomerize in the mitochondria outer membrane and induce MOMP. Multi-domain anti-apoptotic proteins such as B-cell CLL/lymphoma 2 (BCL2), BCL-XL, BCL-W and MCL-1 prevent MOMP by interacting with and sequestering the multidomain pro-apoptotic Bcl proteins. BH3-only proteins form the largest subfamily of BCL2 proteins that can facilitate MOMP by two mechanisms: via direct interaction with BAX and BAK or indirectly by competitive binding to multi-domain anti-apoptotic proteins and preventing their interactions with BAK and BAX [30]. 

Each BH3-only protein has a unique profile of binding partners. Thus, BAD has been shown to bind to and neutralize BCL-2, BCL-XL, and BCL-W, displacing BAK and BAX and promoting pore formation. Phosphorylation of BAD at S112 (S75 in human BAD) creates binding sites for 14-3-3 chaperons that sequester BAD away from BCLXL, BCL2 and BCL-W, whereas phosphorylation at S155 within BH3 domain disrupts BAD interactions with anti-apoptotic BCL2 family proteins. Therefore, availability of BCLXL to interact with pro-apoptotic proteins and inhibit apoptosis is regulated indirectly via BAD phosphorylation [31]. However, other anti-apoptotic proteins such as MCL-1 and A1 are not neutralized by BAD, but instead are bound to and neutralized by Noxa and p53 upregulated modulator of apoptosis (PUMA), respectively [32,33,34,35]. 

Unlike BCLXL, MCL-1 is characterized by a rapid turnover [36,37]. Expression and anti-apoptotic function of MCL-1 is regulated at transcriptional, post-transcriptional and post-translational levels by several signaling mechanisms. Depending on cell type, MCL-1 transcription can be increased by epidermal growth factor (EGF), vascular endothelial growth factor (VEGF), interleukin (IL)7, IL6, IL5 and granulocyte-macrophage colony-stimulating facror (GM-SCF). The promoter region of MCL-1 contains binding sites for signal transducer and activator of transcription (STAT), cyclic AMP response element (CRE) and NFκB transcription factors, and binding of STAT3, STAT5, cAMP response element binding protein (CREB), specificity protein 1 (SP1) and hypoxia-inducible factor 1 alpha HIF1α to MCL-1 promoter have been documented [36,37]. At post-transcriptional level MCL-1 mRNA stability is increased by mir29 binding to 3′UTR, whereas alternative splicing produces shortened functionally impaired forms of MCL-1 that cannot interact with pro-apoptotic proteins of Bcl2 family. At post-translational levels, the expression of MCL-1 is regulated by ubiquitin-dependent degradation by 26S proteasome. A MULE/LASU1 and beta-TrCP were identified as E3 ubiquitin ligases responsible for rapid turnover of MCL-1 in a variety of cell types whereas USP9X deubiquitinase stabilize MCL-1 levels. Phosphorylation provides yet another level of regulation of MCL-1 stability. Thus, phosphorylation at Thr163 by ERK, glycogen synthase kinase 3 (GSK3) or c-Jun N-terminal kinase (JNK) primes MCL-1 for additional phosphorylations at S155 and S159 by GSK3 that increase MCL-1 turnover, or for phosphorylations at S121 by JNK, and p38 as well as at T92 by ERK1 that stabilize MCL-1. Activation of PKA signaling has been connected with stabilization of MCL-1 in tissue culture and in vivo cancer models [38,39]. 

It has been shown that ADRB2/PKA signaling can phosphorylate BAD and dissociate it from BCL-XL and also upregulate MCL-1 levels in prostate cancer cells [27,29,40]. Thus, ADRB2/PKA signaling targets two arms of BCL2 network by increasing relative levels of both BCLXL and MCL-1, which results in more efficient apoptosis inhibition comparing to signaling pathways that target a singular arm. 

In addition to direct effects on prostate cancer cells, activation of ADRB2 influences tumor growth indirectly by stimulating angiogenesis. Thus, ADRB2/PKA/CREB signaling increased neovascularization in xenograft models of prostate cancer by sustained epigenetic inhibition of the angiogenesis suppressor thrombospondin-1 TSP1 [41]. NE secreted into the tumor microenvironment by autonomic adrenergic nerves increased density and branching of capillaries in prostate tumors by activating ADRB2 signaling in endothelial cells [42,43].

**Figure 1:**
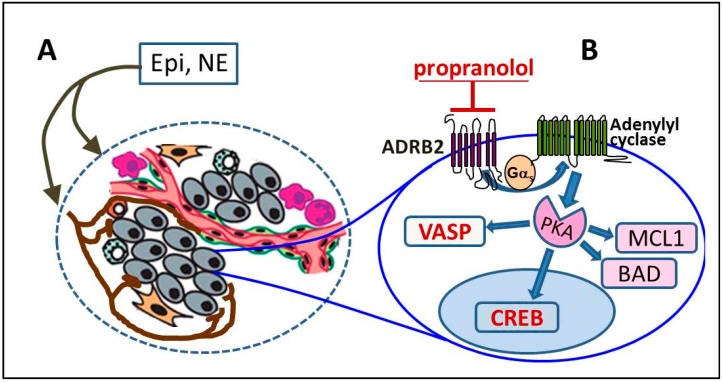
ADRB2 signaling in prostate cancer. (**A**) epinephrine (Epi) and norepinephrine (NE) from circulation and secreted locally by sympathetic nerves, neurodifferentiated prostate cancer cells and macrophages activate anti-apoptotic signaling in prostate cancer cells and stimulate angiogenesis and invasion; (**B**) Activation of ADRB2 signaling (that can be inhibited by propranolol) induces phosphorylation of PKA substrates pS133CREB, pS157VASP, p75BAD and increases expression of myeloid cell leukemia 1 (MCL-1). Dephosphorylations of vasodilator-stimulated phosphoprotein (VASP) and cAMP response element binding protein (CREB) reflect inactivation of β-2 adrenergic receptor/cAMP-dependent protein kinase (ADRB2/PKA) signaling pathway by propranolol, whereas decreased levels of MCL-1 and p75BAD predict whether propranolol will sensitize prostate cells to apoptosis.

Mechanistic studies summarized above suggest a model that connects ADRB2 signaling with PC (Figure 1). In brief, Epi and NE from circulation and secreted in tumor microenvironment by nerve terminals, neurodifferentiated prostate cells and by stroma immune cells contribute to prostate cancer progression and therapy resistance by inhibiting apoptosis, accelerating invasion and neovascularization. Chronically elevated catecholamines induce neuroendocrine differentiation of prostate epithelial cells. Secretion of Epi/NE by immune cells and by neurodifferentiated prostate cells could trigger a vicious circle of sustained ADRB2 signaling in prostate tumors.

The role of beta-adrenergic signaling in prostate cancer has been assessed in several retrospective epidemiological studies that examined effects of β-blockers on prostate cancer incidence and mortality. The initial evidence implicating beta-adrenergic signaling in prostate cancer progression came from a Canadian study showing 18% decrease in prostate cancer incidence among users of β-blockers [44]. This observation was supported by another retrospective study of 3561 patients with high risk prostate cancer or metastatic disease, which showed extended survival for patients who took β-blockers (hazard ratio (HR): 079; confidence interval (CI): 0.68–0.91; *p* < 0.001) [45]. Recently, decreased mortality among β-blocker users across multiple cancers (including a 20% reduction in mortality from male reproductive neoplasms) was reported in an analysis of the US FDA Adverse Events Reporting System [46]. However, other studies found no connection between β-blockers and prostate cancer [47,48]. There were also concerns that decreased mortality from prostate cancer in β-blocker users is due to the increased mortality from other causes rather than extended cancer survival [49]. 

The interpretation of these retrospective studies is complicated because they did not discriminate between β-1 selective blockers like atenolol that primarily inhibit ADRB1 receptors and 13 fold less efficient in inhibiting ADRB2 signaling, and propranolol that inhibits both ADRB1 and ADRB2 receptors [17]. Mechanistic studies in preclinical models unequivocally demonstrated the role of ADRB2 rather than ADRB1 signaling for prostate cancer progression and therapy resistance. Therefore, propranolol, but not β-1-selective blockers, would be expected to have effect on prostate cancer. Indeed, a retrospective study of 12,119 patients who took propranolol showed a significant decrease in the incidence of several cancers including prostate cancer (HR: 0.52; CI: 0.33–0.83; *p* < 0.01) [50]. Taken together, these findings strongly suggest that ADRB2 signaling contributes to prostate cancer progression and resistance to therapy. Conversely, ADRB2 blockade may extend the survival of PC patients. 

Propranolol is a clinically approved antagonist of ADRB1 and ADRB2 prescribed to treat cardiovascular diseases, anxiety and related disorders [51,52]. Propranolol pharmacodynamics and contraindications are well established; therefore, if the benefits of propranolol are demonstrated in clinical trials it can be repurposed for treatment of PC in combinations with existing therapies. To assess the therapeutic potential of propranolol for prostate cancer prospective clinical trials are needed that focus on patients with active ADRB2 signaling. Side effects of propranolol include impotence, bradycardia and hypotension. To avoid unnecessary risks, patients without active ADRB2 signaling or with active mechanisms that can render ADRB2 blockade inefficient should be excluding from clinical trials of propranolol. 

Selection of patients for propranolol clinical trials should be guided by biomarkers and classification strategies based on analysis of interactions between ADRB2 signaling and other signaling mechanisms that contribute to PC pathogenesis. The rationale for identification of biomarkers to select patients for propranolol trials is discussed below. 

## 3. Identifying Tumors with Active ADRB2 Signaling

Epi is an effector of Hypothalamic-Pituitary-Adrenal (HPA) axis released systemically by adrenal cortex in response to psycho-emotional, metabolic or physical stress [53]. Increased stress and anxiety have been reported among prostate cancer patients [54,55,56,57]. Experiments in prostate cancer cells show that 1nM of Epi is sufficient to activate ADRB2/PKA pathway and induce phosphorylation of PKA substrates pS133CREB and pS75BAD [40]. Consistent with these tissue culture data, phosphorylation of pS133CREB and pS75BAD is detected in prostates of mice subjected by immobilization stress or injected with Epi. 

In pilot clinical studies increased levels of Epi were detected in 20% of plasma samples collected from PC patients [27] and a highly significant positive correlation (0.91; *p* < 0,0001) was observed between increased blood Epi and the phosphorylation of S133CREB in prostate biopsies [58], which supports the relevance of preclinical model data to human prostate gland. However, no correlation between self-assessed psycho-emotional stress levels and plasma Epi has been found [27]. Therefore, longitudinal studies of plasma catecholamies in prostate cancer patients are needed to determine if a group of prostate cancer patients exist with continuously elevated catecholamines and whether these patients can be identified based on stress questionnaires or by biochemical tests. 

NE is another ADRB2 ligand locally secreted in prostate gland primarily by sympathetic nerve terminals and to a lesser extent by macrophages and possibly prostate epithelial cells transdifferentiated into neuroendocrine cells. Prostate is highly innervated by sympathetic nerves, and this innervation is required for prostate organogenesis and maintenance after puberty [59]. Sympathetic nerves fire during ejaculation and stimulate secretion by prostate epithelial cells [60]. In the mouse models of prostate and ovarian cancers immobilization stress increased both Epi and NE in cancer xenografts, and prostate glands [27,61]. Increased urine NE but not Epi was reported in metastatic cancer patients [62]. NE-induced activation of ADRB2 signaling could be particularly relevant for prostate cancer with neuroendocrine differentiation and especially for castration resistant neuroendocrine prostate cancer (CRPC-NE) [5,63]. Initially considered infrequent, CRPC-NE is now recognized as more prevalent, especially among patients with castration resistant disease. Described types of CRPC-NE include: conventional adenocarcinoma of the prostate with focal neuroendocrine differentiation; carcinoid and carcinoid-like tumors; and small cell undifferentiated neuroendocrine carcinoma of the prostate [63]. It is unknown whether patients diagnosed with CRPC-NE show increased NE levels in prostate tumors and activation of ADRB2. 

In summary, NE locally secreted in prostate gland and in prostate tumor microenvironment may play comparable or even more important role than Epi in activation of ADRB2/PKA signaling in prostate cancer cells. It is not clear, however, whether increased local NE concentrations in prostate tumors would lead to systemic increases in NE that can be detected by measuring plasma or urine NE. In fact, no correlation between increased NE in tumors and systemic NE levels was detected in ovarian cancer patients [64,65]. Conversely, it is unknown if increased plasma NE predict activation of ADRB2 signaling in normal prostate tissue and prostate tumors. 

Increased levels of plasma NE have been reported in patients with post-traumatic stress disorder (PTSD) and untreated hypertension [66,67,68]. Analysis of prostate tumors for NE and ADRB2 activation in this group of patients with elevated plasma NE will help to clarify relationships between systemic NE, tumor NE and ADRB2 activation. 

In addition to increased systemic and local concentrations of catecholamines, enhanced activation of ADRB2 signaling pathway could be a result of impaired down regulation mechanisms that can take place at each step of ADRB2→adenylyl cyclase→PKA signaling cascade. ADRB2 desensitization involves PKA and protein kinase C (PKC) activated downstream from Gα_s_ and more recently discovered specialized G protein-coupled receptor kinases (GRK) activated downstream from Gβγ. Phosphorylation of cytoplasmic loops of ADRB2 by PKA and PKC “uncouples” ADRB2 from Gα_s_ and may switch receptor specificity from Gα_s_ to Gα_i_; whereas phosphorylation of cytoplasmic loops of active ADRB2 by GRKs promotes interactions with beta-arrestins that sterically inhibit interaction between ADRB2 and Gα and lead to ADRB2 internalization and degradation [11]. Yet another long-term downregulation mechanism of ADRB2 signaling involves inhibition of ADRB2 mRNA transcription [69,70]. Expressions of BARK1/GRK2 and ADRB2 mRNA are reportedly decreased in advanced CRPC [21,71]. However, it is not clear if these changes are associated with constitutive activation of signaling pathways downstream from ADRB2 as systematic analysis of ADRB2 signaling in prostate cancer have not been performed. 

In summary, existing data from preclinical and clinical studies suggest that increased plasma Epi can serve as reliable criterion to identify patients with active ADRB2 signaling in prostate tumors. Additional studies are needed to determine if activation of ADRB2 signaling occurs in patients with increased plasma NE and in patients diagnosed with CRPC-NE. Considering that other factors besides increased catecholamines (impaired downregulation due to the loss of BARK1, for example) may lead to activation of ADRB2 prostate tumors, analysis of biopsies for levels of catecholamines and phosphorylation of PKA substrates remains the most reliable method to identify patients with active ADRB2 pathway. 

Future clinical studies that examine levels of Epi and NE in plasma and in tumors together with the activation of PKA signaling in tumors of patients who take or do not take propranolol will inform whether activation of PKA pathway is a reflection of catecholamine-induced ADRB2 signaling in prostate tumors and whether propranolol can inhibit this activity. 

## 4. Identifying Prostate Tumors Unresponsive to Propranolol

**Figure 2:**
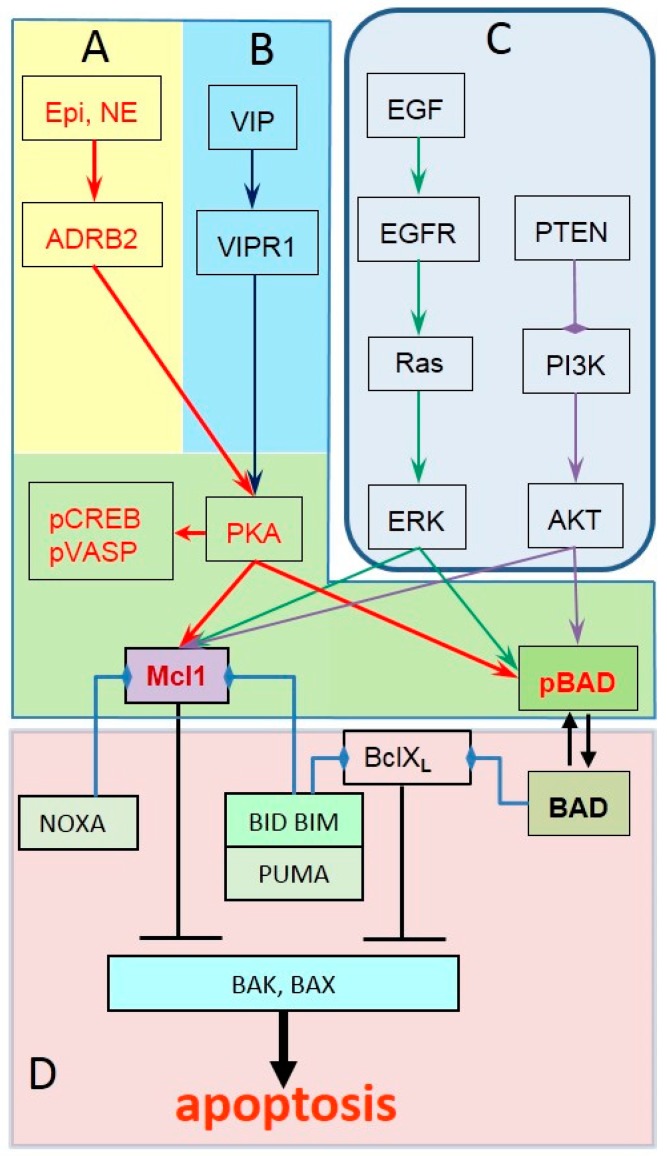
Anti-apoptotic signaling pathways in prostate cancer converge on MCL-1 and BAD. (**A**) Epi and NE activate ADRB2/cAMP/PKA signaling cascade that in turn phosphorylates S133CREB, S157VASP, S75BAD and increases MCL-1 expression; (**B**) Other Gα_s_ coupled GPCRs can activate cAMP/PKA and induce similar pattern of phosphorylated PKA substrates as ADRB2 activation; (**C**) GPCR-independent signaling pathways (Receptor tyrosine kinases, Ras; PI3K/AKT) can induce S75BAD phosphorylation and increase expression of MCL-1; (**D**) Expression of BclXL and/or loss of NOXA, BIM, PUMA can increase apoptosis threshold without changing BAD phosphorylation or MCL-1 levels.

To supplement a set of criteria that identify potential responders (increased plasma Epi, NE and phosphorylation of PKA substrates in prostate tumors), additional classifiers can be defined to exclude patients who are unlikely to benefit from propranolol. Based on the mechanisms of propranolol unresponsiveness, prostate tumors can be divided into four groups (Figure 2). 

First, tumors that do not show activation of ADRB2 signaling and rely on other mechanisms to sustain growth and survival. The absence of phosphorylation of PKA substrates in tumor biopsies is a strong indication against propranolol therapy.

Second, tumors in which activation of PKA may occur independently from ADRB2. Indeed, prostate epithelial cells express other GPCRs coupled to Gα_s_ (PTH1R, CALCR, VIPR1 etc.) that can render cAMP/PKA signaling cascade insensitive to propranolol. For example, activation of calcitonin and vasoactive intestinal polypeptide (VIP) receptors have been shown to support androgen-independent growth of prostate cancer cells [72,73,74]. Similarly to NE, VIP is secreted by autonomic nerves in the prostate gland and also can be produced by prostate cancer cells [75,76,77]. In the same way as ADRB2, VIP receptors VIPR1 and VIPR2 are expressed in prostate cancer and engage PKA/pS75BAD mechanism to inhibit apoptosis in prostate cancer cells [78,79]. Sustained phosphorylation of PKA substrates after propranolol therapy would be an indication for activation of adenylyl cyclase and downstream signaling by ADRB2-independent mechanisms (Figure 2A,B).

Third, tumors in which S75BAD phosphorylation and expression of MCL-1 (that were identified as targets of ADRB2/PKA signaling responsible for anti-apoptotic effects of ADRB2 activation) are controlled by other signaling mechanisms. However, beside ADRB2/PKA other signaling mechanisms may control these molecules. Thus, PI3K/AKT EGFR/ERK and TNFα/IKK pathways are known to phosphorylate S75BAD and increase MCL-1 expression [28,37,80]. As a result, increased expression of MCL-1 and BAD phosphorylation could be accomplished by via other propranolol-insensitive pathways when ADRB2/PKA signaling pathway is inhibited. Tumors is this category are expected to show decreased phosphorylation of PKA substrates (pS133CREB, pS157VASP) but sustain BAD phosphorylation and MCL-1 levels after propranolol therapy (Figure 2C). 

Fourth, in a broader context, the effects of propranolol on apoptosis in prostate cancer cells will also depend on other BCL family proteins beside BAD and MCL-1. Sensitivity to apoptosis induced by BAD dephosphorylation and loss of MCL-1 expression varies among prostate cancer cell lines as does the expression of anti- and pro-apoptotic BCL proteins. For example, as compared to C42 cells, PC3 and DU145 cells are less sensitive to apoptosis due to increased expression of BclXL or loss of BAX expression, respectively. Knockdown of BCL-XL lowered the threshold for apoptosis induced by agents that trigger BAD dephosphorylation, whereas knockdown of BIM and PUMA decreased apoptosis induced by agents that downregulate MCL-1 [81,82]. Analysis of mRNA from metastatic and hormone resistant prostate cancer demonstrated increased expression of BclXL as compared to normal tissue and primary tumors, while there was no significant differences in Bcl2 and BAX expression between these groups [83]. Consistent with these findings, immunohistochemical analysis of BCL-2, BAX, BCL-XL, and MCL-1 in primary low grade and advanced grade prostate tumors showed similar patterns with substantial increase in MCL-1 and BclXL immunoreactivity in high grade metastatic tumors and modest increase in BCL-2, while no significant changes were reported for BAX [84]. Yet another study reported significant increase in positive staining for BCL-2 (1% vs. 11%) in metastatic tumors [85]. Tumors in this fourth category with changed expression of BCL-2 family proteins will show decreased MCL-1 expression and BAD dephosphorylation in response to propranolol but will not increase apoptosis (Figure 2D). 

In addition to regulatory molecules that define apoptosis sensitivity of prostate cancer cells, ADRB2 signaling may stimulate prostate cancer progression by altering expression of angiogenesis regulators VEGF1 and TSP1 [41,43]. Similarly to apoptosis regulation, other signaling mechanisms unrelated to ADRB2 can control angiogenesis in prostate tumors. Thus, TSP1 and VEGF levels can be also considered as potential biomarkers to predict tumor response to propranolol. 

## 5. Conclusions 

Experiments in mouse models of prostate cancer and pilot clinical studies show that ADRB2 is an integral part of highly redundant network of signaling pathways that contribute to PC progression and therapy resistance. Analysis of ADRB2/PKA signaling confirmed that targeted apoptosis regulatory molecules are not unique for this pathway, but instead, are shared with other signaling mechanisms that operate in advanced prostate cancer. Therefore, topology of signaling network that include ADRB2/PKA pathway should be considered when clinical trials of propranolol are designed. 

To produce conclusive results, clinical trials of propranolol should use a personalized approach with stringent criteria to select patients that are expected to benefit from propranolol therapy. Active ADRB2 signaling defined by increased systemic Epi and/or increased Epi/NE in the tumors together with activation of PKA signaling pathway (phosphorylation of PKA substrates S133CREB, S157VASP and S75BAD) should be the main inclusion criterion, whereas patients that do not show activation of ADRB2/PKA pathway should be excluded. Patients who receive propranolol should be further evaluated for biomarkers of PKA activity (pS133CREB, pS157VASP) apoptosis sensitivity (pS75BAD and MCL-1) and perhaps angiogenesis (TSP1 and VEGF) as well. In the absence of changes in the phosphorylation and expression levels of these biomarkers, the expediency of propranolol monotherapy therapy should be reassessed, and the status of other signaling pathways should be evaluated. Analysis of prospective propranolol clinical trials data for correlations between progression free survival, overall survival and changes in the biomarkers of ADRB2/PKA activity, apoptosis and angiogenesis will inform whether biomarkers discussed in this review can segregate prostate cancer patients into propranolol responsive and nonresponsive groups.

Although the focus of this review is inhibition of ADRB2 signaling by propranolol, it is apparent that future progress toward efficient PC therapy will depend on combinations of inverse ADRB agonists with inhibitors targeting redundant signaling pathways that converge on critical effector molecules that control apoptosis, metabolism and angiogenesis in prostate tumors.
